# The Treatment of Some Phases of Alveolar Abscesses

**Published:** 1905-06-15

**Authors:** Geo. W. Cook

**Affiliations:** Chicago


### THE TREATMENT OF SOME PHASES OF ALVEOLAR ABSCESSES.

BY GEO. W. COOK, B.S., D.D.S., CHICAGO.

Read before the Southwestern Michigan Dental Society, April 11, 1905.

In the treatment of any disease process, and especially any local inflammatory condition with or without the tendency of pus formation, much depends upon our conceptions with reference to the stage of tissue changes. The kind of tissue that is involved in the process, the amount and quality of the exciting cause, a full recognition of the phenomena of predisposing certain tissue to be acted upon, and the virulent properties of the bacteria that enter such tissue will greatly influence treatment.

The tissue from an anatomical and histological point of view that is involved in an alveolar abscess, as well as its physiological function, is endowed with certain protective tendencies which give considerable power of immunity, and, at the same time, a destructive influence on bacteria themselves. At the present time we are conversant with the fact that the general physiologic condition of an individual may play an important role in bringing about a predisposition to certain local inflammatory processes, as well as to produce changes in the organisms that find a habitat in the oral cavity.

There are many persons who suffer from chronic alveolar abscesses, which, in the majority of instances, results in a fistulous opening, and the broken down tissue cells are discharged into the oral cavity and become one of the constituents of the fluids of the mouth, which may contaminate this secretion in such a way as to prevent its physiological action on the food that is being introduced into the stomach and intestines for the purpose of replenishing the waste products of the body. The condition herein mentioned may not bring about an appreciable change in the general constitution for a considerable period, but it is well known that the gastric juice possesses marked antiseptic and germicidal properties, and the bacteria that pass into the stomach by way of the saliva may be so acted upon by the gastric juice that their effects may not be observed until the individual finds himself in the susceptible condition for other and more important diseases. Therefore, persons suffering from chronic alveolar abscesses, such as has just been mentioned, are always more or less liable to some constitutional disturbances in which it would be quite difficult to trace the initial cause to this almost unrecognized condition of chronic alveolar abscess.

Therefore, it is the duty of the practicing dentist to constantly bear in mind that upon his efficiency and skill in treating the teeth preparatory to crowning and filling may possibly depend the value of a healthy and useful life to the individuals who are receiving treatment at his hands. A large majority of these chronic alveolar abscesses are the result of improper treatment of the teeth preparatory to crowning and filling.

Chronic inflammatory conditions in the tissue, such as are located in the apical ends of the roots of teeth, is usually brought about by a continuous, mild, irritating action by a form of bacteria which possess but small virulent properties, and are sometimes almost incapable of setting up a process of sufficient activity as to establish a fistulous opening, but whose activity results in the degeneration of the tissue cells around the foci of irritation. In such cases we usually have what is designated the blind chronic alveolar abscess. This mild degenerative condition of the tissue may continue for some time with only a slight soreness of the tooth, but suddenly, and at some unexpected time, it assumes a process more or less acute in its pathologic changes, with a soreness and more or less uncomfortable feeling when the jaws are brought in occlusion with each other. On the other hand this mild disturbance may continue for a long time, following a certain line in the cancellated tissue of the jaw until it has formed a considerable cavity either around the apical end of the root, or it may assume a direction in the tissue following the lymphatic structures until it has destroyed considerable tissue, and has assumed a position where it is impossible for the tissue to resist the breaking down of its parts, The bacteria then take on a more virulent nature and enter into the lymphatic circulation and are possibly carried to a remote part of the body, there assuming a more destructive and detrimental influence to the tissue of that vicinity.

The treatment of such conditions as above mentioned must always vary in accordance with the extent of the destructive process and with the constitutional conditions of the patient at the time he presents himself for treatment. One of the very essential things in such cases is to gain access through the root of the tooth and determine, so far as possible, the extent of the lesion, especially if there be no fistulous opening. When this initial mechanical process has been attained the question that first presents itself is, How and what agents can best be used? One of the conditions that must be avoided as far as possible is the establishment of an acute inflammatory condition, for the simple reason that if there is an extended tissue degeneration in the locality of the abscess, there will be more or less pain and a far greater destruction of the tissue than should occur if the usefulness of the tooth is to be taken into consideration. Then it is essential that we avoid the introduction of a strong escharotic agent or those that are volatile in their tendencies. First the escharotic agent will cause a destructive change in the tissue that may be still in possession of sufficient physiological function to regenerate itself, provided these irritating agents which are present in the form of bacteria are arrested in their activity for a sufficient time to enable the tissue to re-establish themselves or neutralize the poisonous agents that are present as the result of the action of bacteria on the tissue. If a volatile agent be introduced, and especially if it is confined by sealing up the cavity in the tooth, as the process of volatilization of the drug goes on it will carry with it many of the inactive bacterial cells into the tissue which has but little power of resistance, and thus establish a more active process in the tissue and in a great many instances cause an intense reaction of the tissue, which will bring about an acute form of disease which, so far as possible, should be avoided.

In this connection it may be well to mention that the regenerative processes of the bony structure is never as rapid as where regeneration of tissue takes place in the soft tissues. Bone regeneration is established by a certain morphological re-arrangement of the cells and goes on for a considerable time after apparently a normal physiological condition has been established. The period between the elimination of the bacterial irritant and the re-establishment of the normal processes is a period when teeth should be allowed to rest. The operations of filling with gold or fitting bands for crowns, etc., should be postponed for at least two or three weeks after a severe, acute or even a chronic alveolar abscess has been restored to a normal physiological function. The parts should be allowed to rest for a sufficient length of time that the tissues may re-adjust themselves to a normal mechanical resistance to irritating agents that are not infectious in character, and all agents used for the purpose of treating these conditions should possess the quality of slightly stimulating without irritating the tissue cells, and inhibit the development of the bacteria.

The profession of dentistry is constantly having brought to its notice agents that are supposed to be antiseptic and germicidal, and which are recommended for filling root canals to remain there as permanent antiseptics. Carbolic acid is one of the agents that the profession has used in cases of chronic abscesses with fistula, and many claim it to be superior to anything they have tried in the treatment of such conditions; possibly there is no agent that can come as near being universally used by the inexperienced in fistulous abscesses, but there are many agents that are quite superior if we will but study the pathological conditions we have to treat. In cases of abscesses where there is only a fistulous tract without much tissue degeneration around the apical end of the root, an agent that I have tried with considerable satisfaction is guaiacol carbonate, which is a combination of guaiacol and carbolic acid. This agent has antiseptic properties sufficient to destroy the form of bacteria that is usually involved in such processes; it has the beneficial effect on the tissue of being but little escharotic and is a mild stimulant to the tissue cells.

In the treatment of these cases, where a mild disinfectant is required and tissue stimulation is indicated, this agent should have a valuable place in the treatment of these conditions. It is valuable also in the treatment of some forms of antrum troubles.

In the so-called blind abscesses, we mean those where there is no fistulous opening, as we have previously stated, it is important that these conditions be treated in a different manner from those with a fistulous opening. The guaiacol carbonate in such pathological conditions as a blind abscess would, unless carefully handled, prove to be slightly irritating. We have observed that when this agent is confined in a place like that of the root of a tooth and at body temperature, that it becomes sufficiently volatile in some cases as to produce the very condition that we wish to avoid, namely, acute inflammatory process. In blind abscesses where there is but little destructive change in the tissue at the apical end of the root, and where slight discoloration of the tooth is of no special importance, there is probably no agent that will give such general satisfaction as that of a ten or fifteen percent solution of chinosol. But as I have elsewhere stated, this agent must be handled with the greatest of care if discoloration of the tooth structure is to be avoided. Chinosol has the distinctive value of being extremely germicidal when brought into contact with such bacteria as are usually involved in chronic alveolar abscesses, especially where there is a slight but continuous degeneration of the tissue and where the bacteria are but slightly pathogenic in their tendency. This agent may also be said to be beneficial in cases of antrum trouble, where there is but little pus formation; for instance, in those case where there is a mucous degeneration of the tissues following a catarrhal inflammation. Chinosol, when introduced into large pus cavities, is rapidly neutralized by the pus, consequently its effects are not as beneficial in large pus cavities as that of the guaiacol carbonate.

